# Molecular Oxygen Levels and Percentages of DNA Damage in TPN Patients

**DOI:** 10.3390/nu15092206

**Published:** 2023-05-06

**Authors:** Karolina Dąbrowska, Zuzanna Zaczek, Mariusz Panczyk, Sylwia Osowska, Paweł Kowalczyk, Karol Kramkowski, Jacek Sobocki

**Affiliations:** 1 Department of General Surgery and Clinical Nutrition, Centre of Postgraduate Medical Education, 01-813 Warsaw, Poland; 2 Department of Human Nutrition, Faculty of Health Sciences, Medical University of Warsaw, Erazma Ciołka 27, 01-445 Warsaw, Poland; 3 Department of Education and Research in Health Sciences, Faculty of Health Sciences, Medical University of Warsaw, Litewska 14/16, 00-581 Warsaw, Poland; 4 Department of Applied Pharmacy, Warsaw Medical University, Banacha 1, 02-097 Warszawa, Poland; 5 The Kielanowski Institute of Animal Physiology and Nutrition, Polish Academy of Sciences, 05-110 Jablonna, Poland; 6 Department of Physical Chemistry, Medical University of Bialystok, Kilińskiego 1, 15-089 Bialystok, Poland

**Keywords:** parenteral nutrition, lipids emulsion, liver damage, molecular oxygen, DNA damage

## Abstract

Total parenteral nutrition (TPN) is a life-saving therapy for patients with intestinal failure, but it carries the risk of complications, including an increase in liver enzymes alanine aminotransferase (ALT) and aspartate aminotransferase (AST) after long-term use. Patients receiving chronic TPN are also exposed to metabolic stress from both the underlying disease and parenteral nutrition. The aim of this study was to compare the concentration of liver transaminases AST and ALT in relation to the rate of oxygen consumption in platelet mitochondria in patients receiving long-term TPN with the degree of oxidative stress induced by lipid emulsions, and to explain their role in cellular energy metabolism and changes in the liver based on the percentage of genomic DNA damage. The study group consisted of 86 TPN patients, while the control group consisted of 86 healthy volunteers who were fed only orally. The results of the study showed that the percentage of molecular oxygen depended on the type of lipid emulsion supplied. Analyzing time on TPN as a factor, we observed a decrease in percentage genomic DNA damage and an increase in percentage molecular oxygen in cells. It remains unclear whether TPN has a direct effect on genomic DNA damage and the level of molecular oxygen in cells during the course of treatment. In conclusion, this study provides important insights into the potential effects of TPN on liver enzymes and cellular metabolism. Further research is needed to better understand the underlying mechanisms and to develop strategies to minimize the risk of complications associated with TPN.

## 1. Introduction

Total parenteral nutrition (TPN) has significantly improved the quality of life and survival of patients who cannot be fed orally [1,2]. In this procedure, vital nutrients are delivered intravenously. Despite many medical benefits, TPN can cause progressive organ damage, including liver damage. Levels of the liver enzymes alanine transaminase (ALT) and aspartate transaminase (AST) often increase with long-term use of TPN [3].

Patients receiving chronic parenteral nutrition are subject to metabolic stress from both the underlying disease and parenteral nutrition. TPN may affect gene expression in the liver and cause oxidative disorders. Increased oxidative stress (OS) can affect cell structures and potentially impact tissues; as diseases progress, oxidative damage to nuclear and mitochondrial DNA (deoxyribonucleic acid) escalates. Mitochondria are highly susceptible to oxidative damage. Reactive oxygen species (ROS) can attack the cell membrane and alter the intermembrane potential of mitochondria, reducingintegrity [4]. ROS can also cause mutations in mitochondrial DNA [5]. The role of mitochondria is to produce ATP (adenosine triphosphate) in the process of cellular respiration and to regulate cellular metabolism [6]. Decreased ATP production in mitochondria often occurs in severe diseases [7]. Since the maintenance of proper mitochondrial function depends on certain micronutrients, it is very important to provide the body with an adequate amount of nutrients [8]. Lipid peroxidation is a complex-free radical reaction triggered by reactive oxygen species, leading to the formation of lipid hydroperoxides and their degradation products [7]. The products of lipid peroxidation can disrupt the flow of electrons along the respiratory chain, leading to the excessive reduction of respiratory chain components and an increase in reactive oxygen species in mitochondria. In a healthy organism, there is a balance between the production of ROS and antioxidant defense. The release of ROS, which consist of oxygen free radicals and other chemicals, can deplete antioxidant systems. They interact with proteins, lipids, and nucleic acids and irrevocably destroy or alter the functions of body cells [9]. ROS can increase the production of tumor necrosis factor α (TNF-α) and oxidize fat deposits. The cell membrane, which is composed of polyunsaturated fatty acids, is a primary target for reactive oxygen which damages the cell membrane. Polyunsaturated fatty acids such as linoleic and arachidonic acids, which are present in lipid membranes as phosphoglyceride esters, are particularly susceptible to auto-oxidation. This process is associated with pathological events in biological systems, such as cell membrane damage during aging and exposure to certain toxic substances from the environment [10,11]. Omega-3 fatty acids are recognized as hepatoprotective components of parenteral nutrition admixture but may be susceptible to lipid peroxidation, whereas olive oil is considered more resistant to peroxidation [9,12]. Omega-3 fatty acids are thought to have an anti-inflammatory effect by switching from the pro-inflammatory eicosanoids from omega-6 fatty acids to the anti-inflammatory variant from omega-3 fatty acids [9,13,14]. The administration of ω-6 PUFA (polyunsaturated fatty acids) has been associated with inflammation and oxidative stress [13]. Lipid emulsions high in MUFA (monounsaturated fatty acids) are less susceptible to lipid peroxidation [7,8]. Lipid peroxidation affects lipid metabolism in the liver where cytochrome P-450 is a catalyst in the oxidation of lipid-derived aldehydes to carboxylic acids. Cytochrome P-450-mediated metabolism runs in parallel with other aldehyde metabolism and can serve as a substitute mechanism when other high-yield aldehyde elimination pathways are impaired due to disease or toxicity. Major products of lipid peroxidation such as 4-hydroxynonenal (HNE); unsaturated aldehydes such as acrolein, trans-2-hexenal, and crotonaldehyde; and 2,3-epoxy-nonanal epoxide play an important role in cell signaling pathways. It seems that oxidized lipids have a signaling function in pathological situations. They may be pro-inflammatory agonists and contribute to neuronal death under conditions in which membrane lipid peroxidation occurs. Using the mitochondrial example of mitochondrial lipid cardiolipin as an example, which accounts for up to 18% of all phospholipids, up to 90% of fatty acyl chains are unsaturated. The oxidation of cardiolipin may be one of the critical factors that trigger apoptosis by releasing cytochrome c from the inner mitochondrial membrane and facilitating permeability of the outer membrane. The release of cytochrome c activates the proteolytic cascade leading to apoptotic cell death [10,11]. The oxidative damage in the liver is associated with hepatic lipid metabolism and may affect the absorption and transport mechanisms of tocopherol in this organ. In the liver, morphological damage precedes lipid peroxidation and the consumption of endogenous antioxidants. In the kidney and heart, lipid peroxidation and oxidative damage precedes necrosis [9,13]. There are no predictive factors for damage to the liver and other organs resulting from parenteral nutrition. Oxidative damage may be one of the underlying mechanisms of TPN-associated liver dysfunction. We hypothesized that patients receiving parenteral nutrition would have increased levels of oxidative stress. In the human liver, morphological changes may affect the bile duct structure and function in liver transplant patients as a result of oxidative damage associated with ischemia–reperfusion [11]. In experimental models of rat livers, increased peroxidation is secondary to the increased production of O_2_ and H_2_O_2_ in mitochondria [10,15]. The impact of different lipids on various health-related aspects in adult patients receiving TPN is a topic of growing interest. Nevertheless, there are limited data on how they affect oxidative stress and genomic DNA damage in this group of patients.

The aim of this study was to compare the concentration of the liver transaminases AST and ALT in relation to the rate of oxygen consumption in platelet mitochondria in patients receiving long-term TPN with the degree of oxidative stress induced by lipid emulsions and to explain their role in cellular energy metabolism and the changes in the liver based on the percentage of damaged genomic DNA.

## 2. Materials and Methods

The study group consisted of 86 patients (44 women and 42 men) receiving TPN at a Polish referral center. The average age and duration of parenteral nutrition for each gender are presented in Table 1.

Of the patients, 25.58% had a stoma. The initial cause of intestinal failure and subsequently parenteral nutrition was as follows: cancer surgery (33.72%), mesenteric artery embolism (31.39%), traffic accidents (11.62%), postoperative complications (10.46%), Crohn’s disease (3.48%), congenital disease (3.48%), and other (5.81%).

Patients received TPN based on their individual nutritional needs. In total, 72 of the 86 patients received TPN 7 times per week. Patients received different types of lipid emulsions: 38—soybean oil-based emulsions, 33—emulsions containing fish oil, and 12—olive oil-based emulsions. Two patients used mixed lipids, while one patient received an admixture without added lipids. The volume of lipid emulsions and the number of grams of lipids in the TPN per week are shown in Table 2.

The control group consisted of 86 healthy volunteers who were fed only orally and did not receive parenteral nutrition. Blood was taken for analogous tests in both groups. 

During routine quarterly visits to the TPN center that took place between November and December 2019, patients in the study group had blood drawn for standard tests. The AST, ALT, and C-reactive protein collected during these standard tests were used for the following analysis. In addition, mitochondria were isolated from platelets and their degree of damage was determined by the Oxygen Consumption Assay method using Agilent (USA) MitoXpresIntra kits which use porphyrin-based fluorescent probes to measure the consumption of molecular oxygen in the mitochondria in real-time. Genomic DNA was isolated from whole blood and digested with Fpg (formamidopyrimidine-DNA glycosylase) according to the manufacturer’s instructions (New England Biolabs, Ipswich, MA, USA) to confirm the etheno and propano derivatives in DNA bases generated during lipid peroxidation such as 1,N6-ethenoadenine (A), 3,N4-ethenocytosine (C), N2,3-ethenoguanine (G), and 1,N2-ethenoguanine (G) [16]. The tests were performed by two methods: traditional, namelyusing the Sysmex reagent kit according to the manufacturer’s instructions, and by using the Sysmex XN-200 apparatus. The medical laboratory was accredited by PCA No. AM003. The concentrations of liver enzymes AST and ALT were determined by the spectrophotometric method with reagents and equipment from Abott version G75642R04 and an Alinity device. The applied standards belonged to a specific age range from 32 to 86 years.

### 2.1. Oxygen Consumption Assay

Patients in each experimental group had 2 mL of whole blood drawn in heparinized tubes. The blood was mixed carefully to avoid the formation of microspheres, which could reduce the platelet count and affect the results, and placed on ice for 2 h to allow the sedimentation of individual blood cells. After reaching a visible phase boundary, approximately 150 μL of the upper layer containing platelets was collected and transferred to new tubes containing 300 μL of 10 mM dimethyl sulfoxide (DMSO) for cryopreservation. The samples were stored at −20 °C for further analysis. Before analysis, the fixed platelets were centrifuged at 3500× *g* for 5 min at 2 °C to remove the DMSO. The isolated platelets were dissolved in 300 μL of HEPES-buffered Tyrode solution (119 mM NaCl, 5 mM KCl, 25 mM HEPES buffer, 2 mM CaCl2, 2 mM MgCl2, and 6 g/L glucose, adjusted to a final pH of 7.4 with NaOH). The measurement of the extracellular oxygen consumption rate by platelet mitochondria was performed with a filter-based multimode microplate reader (FLUOstar OPTIMA, BMG Labtech, Offenburg, Germany) using MitoXpress-Xtra HS kits (Luxcel Company, Cork, Ireland) according to the manufacturer’s protocol. Fluorescence measurements were performed in 10-min intervals for 90 min at 37 °C under sealed conditions by adding 100 μL of mineral oil on top to limit the oxygen exchange. Tyrode buffer containing 10 μL of the compound MitoXpress-Xtra HS served as a reference. Each sample was analyzed in duplicate. The rate of change of fluorescence signal per minute was calculated for each 10-min interval and an average signal change per minute over the entire 90-min period was calculated for each patient. Fluorescence traces were converted to oxygen concentration profiles following the work of Konieczka et.al. using the following transformation:

[O_2_] (t) = [O_2_] × Ia × (Io − I (t)) I (t) × (Io − Ia)

where [O_2_] a is the oxygen concentration in the air-saturated buffer (235 μM at 30 °C) and I (t), Ia, and Io are the probe fluorescent signal at time t, signal in air-saturated buffer (baseline signal without enzyme), and signal in deoxidized buffer (maximum signal), respectively [17]. The rate of change of dissolved oxygen was then determined from the initial slopes of these concentration profiles.

### 2.2. Estimation of Oxidative Damage Based on Genomic DNA Digestion by Fpg Protein

Mitochondrial DNA was isolated from 2 mL of frozen blood plasma using a New England Biolabs kit (Labjot, Warsaw, Poland) according to the manufacturer’s instructions. The DNA obtained was digested with Fpg protein (New England Biolabs, cat. no. M0240S, 8000 U/mL) as follows: the Fpg protein was diluted 50-fold with 10× NEB buffer (provided by the manufacturer of Fpg protein) and mixed with 100× BSA solution (also provided with the Fpg protein). Next, 8 µL of purified genomic DNA was mixed with 2 µL of Fpg solution and 2 µL of NEBuffer and incubated at 37 °C for 30 min. The control genomic DNA samples (incubated without the tested compounds) and digested and undigested genomic DNA samples (incubated with the tested compounds) were evaluated by 1% agarose gel electrophoresis. The DNA concentration was determined spectrophotometrically by the A260/A280 ratio. The degree of oxidative damage was estimated using Image Quant software.

The use of Fpg protein, also known as 8-oxoguanine DNA glycosylase or formamidopyrimidine [fapy]-DNA glycosylase, is important because it removes a broad spectrum of oxidized and alkylated bases from double-stranded DNA, including 7,8-dihydro-8-oxoguanine (8-oxoguanine), 8-oxoadenine, and unsubstituted and substituted imidazole ring-opened purines introduced into DNA by hydroxyl radicals (e.g., FapyG, FapyA) as well as by chemical carcinogens, including anticancer drugs (e.g., Fapy-7MeG, Fapy-7EtG, Fapy-7aminoethylG, aflatoxin B1-fapy-guanine, 5-hydroxy-cytosine, and 5-hydroxy-uracil). The Fpg protein has two additional activities: (i) the AP-lyase activity that cleaves both 3’ and 5’ to the AP site, thereby removing the AP site and creating a 1-base gap by beta-elimination and (ii) dRPase activity which removes the 5’ terminal deoxyribose phosphate from DNA incised by an AP endonuclease [18,19,20,21].

The results were estimated by comparing samples digested with Fpg or Mpg (Haag) protein to non-digested samples. The percentage cleavage was determined based on the change in forms resulting from the enzymatic activity of the digested sample relative to the DNA-binding Fpg protein. According to data in the literature damage to genomic DNA greater than three percent by the Fpg protein is a clear indication of damage to all oxidized and modified guanines, which occur as 8-oxoguanine, fapy-adenine (FapyG), and fapy-guanine (FapyA) [18,19,20,21].

The research was approved by the Ethics Committee of the Centre of Postgraduate Medical Education (protocol code 98/2021, date of approval 8 December 2021). Informed consent was obtained from all subjects involved in the study.

## 3. Statistical Analysis

**Figure 1:**
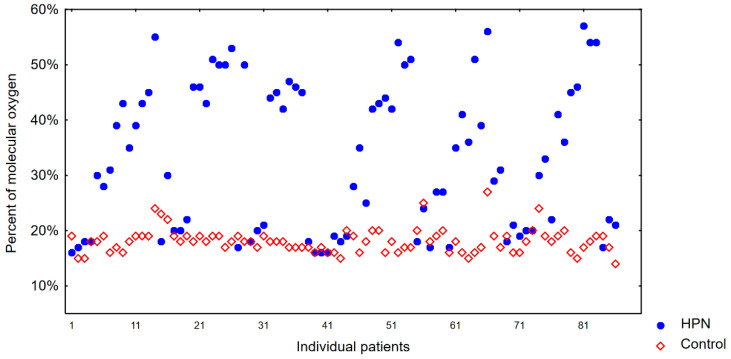
Percentage of molecular oxygen in the control group and TPN group.

**Figure 2:**
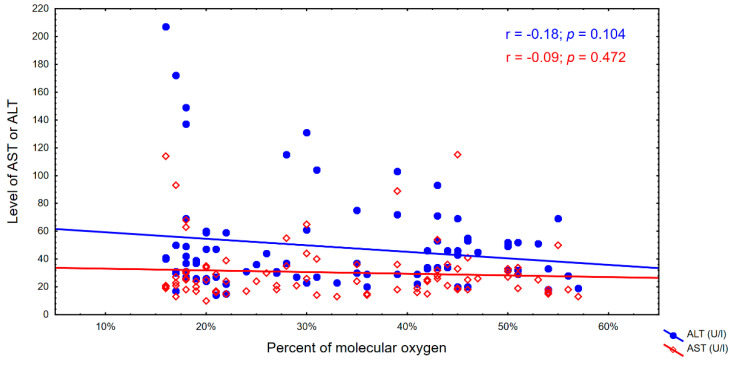
Percentage of molecular oxygen and the AST and ALT levels.

**Figure 3:**
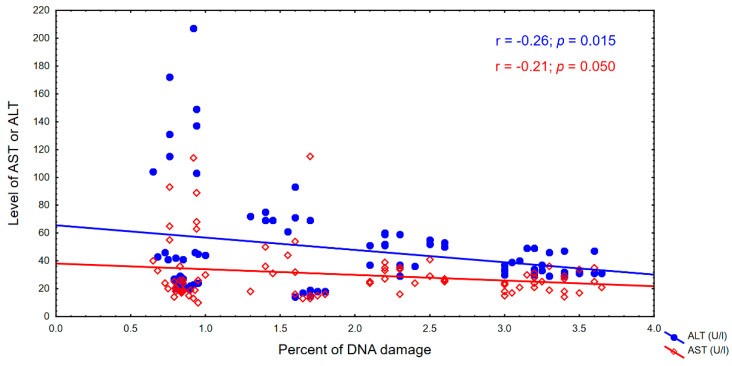
Percentage of DNA damage and the AST and ALT levels.

**Figure 4:**
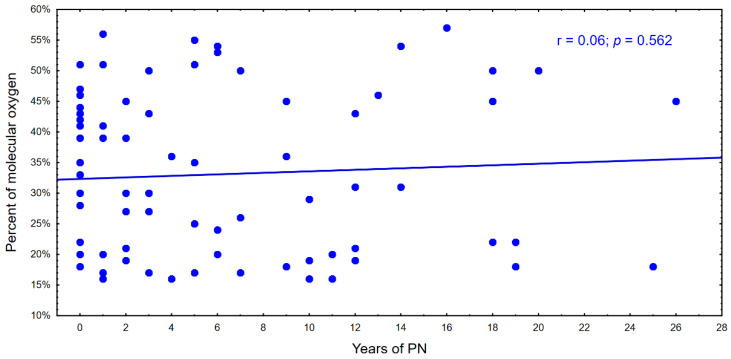
Percentage of molecular oxygen during parenteral nutrition.

**Figure 5:**
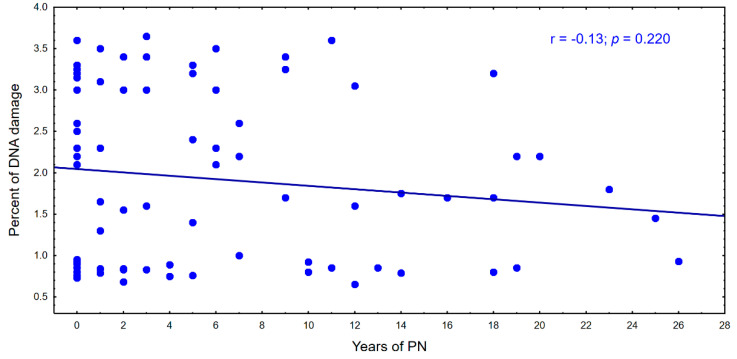
Percentage of DNA during parenteral nutrition.

**Figure 6:**
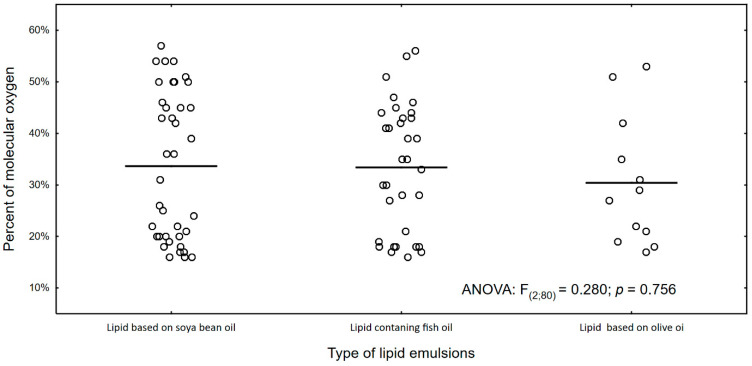
Percentage of molecular oxygen and type of lipid emulsion in PN.

**Figure 7:**
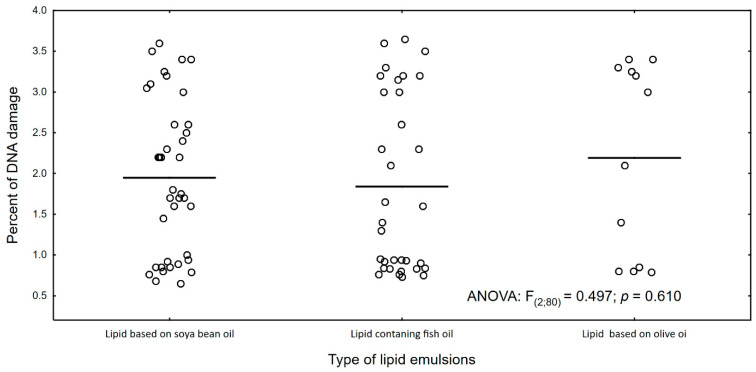
Percentage of DNA damage and type of lipid emulsion in PN.

Variables were presented using descriptive statistics: measures of central tendency and variability (mean (M), standard deviation (SD), number (N), and frequency (%)). The Student’s *t*-test was used to determine a statistically significant difference between the means in two independent samples (women vs. men). The scatter plot method (Figure 1) was used as well as the scatter plot with linear regression lines. This can be seen in Figures 2–5. These plots depict correlations and the correlation coefficient was calculated. Additionally, comparative plots of variability are presented in Figures 6 and 7.

All calculations were made with the use of the STATISTICA™ software package ver. 13.3 (TIBCO Software Inc., Palo Alto, CA, USA). We rejected the null hypothesis and accepted the alternative hypothesis when the *p*-value was less than 0.05.

## 4. Results

In the studied group, a significant increase in the values of AST and ALT was observed in 13.9% of patients. In the remaining cases, the levels of the two liver enzymes were also slightly elevated and deviated from the norm. However, both types of liver parameters studied did not increase the value of the CRP protein. The results were compared with the results of the control group and presented in Table 3.

Increased levels of platelet mitochondrial oxygenation, measured by the oxygen consumption assay, were found in 67.04% of cases. The formation of molecular oxygen-induced damage in platelet mitochondria in the study group was probably caused by their excessive oxygenation and caused the process of hypoxia in the process of oxidative phosphorylation. Hyperoxia was observed in 26.13% of cases. This phenomenon is caused by too low ATP concentrations.

In the studied mitochondrial DNA damage isolated from damaged mitochondria and additionally digested withFpg protein (recognizing oxidized 8-oxoG-type purines and Fapy Ade and FapyGua), the type of mitochondrial DNA damage was found to be closely related to the degree of oxygenation or hypoxia of platelet mitochondria. The extent of genomic DNA damage varied considerably. Levels of damage of 3.5% or more were observed. 

The results of platelet mitochondria oxygenation levels in the study group were compared with those in the control group. The degree of platelet mitochondria oxygenation was 18% in this group and 33% in the study group, indicating that a process of hyperoxia had occurred. The results are shown in Figure 1. 

The activity of ALT was negatively correlated with the percentage content of molecular oxygen, which means that as the activity of this enzyme increases, the percentage content of molecular oxygen in the cell decreases (*p* = 0.024) and the process of cellular DNA damage increases, especially to guanines.

Based on the results of digestion of genomic DNA from the blood of patients with the Fpg protein, damage to the matrix recognizable by the Fpg protein was observed in the form of the so-called fuzzy bands (smear) on agarose gels, indicating a high degree of damage and modification of DNA bases, including modified guanines in the process of the peroxidation of lipids which are new substrates for the analyzed protein. The degree of modification of genomic DNA, which is above 3.5%, proves that very high oxidative stress has occurred in the cell.

Apart from this, increased levels of ALT and AST may indicate liver damage or disease, while a higher percentage of DNA damage caused by Fpg can suggest increased oxidative stress and potential DNA damage.

We have observed an increasing trend in the percentage oxygen content, but it was not statistically significant. There was also a decrease in genomic DNA damage, but this was also not statistically significant. These correlations were observed in both cases, particularly with respect to the ALT indicator. The correlations are shown in Figure 2 and Figure 3.

With the time of parenteral nutrition in the following years, there is a tendency to a decrease in the percentage of genomic DNA damage and an increase in the percentage of molecular oxygen in cells. This relationship is shown in Figure 4 and Figure 5.

Figure 6 and Figure 7 show the percentage of molecular oxygen depending on the type of lipid emulsion supplied. We have observed that the olive oil-based lipid emulsion caused the lowest (average 30%) level of molecular oxygen. However, it causes the highest DNA damage (2.1% on average).

## 5. Discussion

The levels of the liver enzymes ALT and AST often increase with long-term use of TPN [22]. TPN brings many benefits and allows the survival of patients with gastrointestinal insufficiency and those who are unable to deliver adequate amounts of nutrients and minerals through the enteral route. However, this procedure is associated with a high risk of complications due to the use of lipid emulsions. When oxygenation can trigger the so-called oxidative stress and the process of lipid peroxidation, they lead to the development of inflammation in the body [23,24]. Chronic inflammation can promote the development of many diseases and slow down the treatment of existing disorders [23]. Higher levels of fatty acids in the bloodstream promote the deposition of visceral and hepatic fat resulting from decreased lipoprotein synthesis and lipid export from the liver. Subsequently, fatty infiltration of the liver may also cause decreased fatty acid oxidation and fatty liver [25,26,27,28]. Nevertheless, giving up the supply of lipid emulsion would not be beneficial for patients—studies have shown that eicosapentaenoic acid (EPA) and decosahexaenoic acid (DHA) have anti-inflammatory and immunomodulatory properties and that their intake has a positive effect on the health of parenterally fed patients [29,30]. The proper ratio of omega3 to omega6 in the emulsion used to prepare the nutritional mixture also has a beneficial effect [29,30]. In the literature, the relationship between the quantity and quality of the lipid emulsion used and the changes in liver parameters are described in detail [10,31,32,33,34]. Omega-6 fatty acids have an anti-inflammatory effect by suppressing the pro-inflammatory production of cytokines, macrophages, and hepatocytes. They also contribute to the regulation of the synthesis and oxidation of fatty acids in the liver. Their high uptake contributes to increased lipid peroxidation and DNA damage in many cell types, such as macrophages, muscle cells, and liver cells [25,26,27,28]. Due to the high number of double bonds, omega-3 fatty acids are more susceptible to lipid peroxidation and may increase the risk of oxidative stress [35]. Studies by Kosek et al. showed a pro-oxidant state in patients receiving long-term TPN rich in omega-3 [10]. Similar results were obtained by Lavoie et al. in their studies on guinea pigs [36]. Lipid mixtures high in monounsaturated fatty acids are less susceptible to lipid peroxidation. Compared to piglets receiving MCT/LCT (medium-chain triglycerides/long-chain triglycerides), the supply of FO (fish oil) reduced inflammation, promoted fatty acid oxidation, and reduced oxidative stress in the liver [37]. 

AST and ALT may be new potential markers of the oxidative layer, which we can use to estimate not only the degree of damage to the liver after administration of parenteral nutrition but also the damage to mitochondria caused by it. Measuring the oxygen consumption of the cells may facilitate the identification of mitochondrial mechanisms after pharmacological and genetic interventions and determine energy metabolism in relation to the patient’s condition [22,38].

We have observed that the activity of ALT is negatively correlated with the percentage of molecular oxygen. This means that as the activity of this enzyme increases, the percentage of molecular oxygen in the cell decreases (*p* = 0.024), and the process of cellular DNA damage, especially to guanines, increases. This is consistent with previous observations in this type of experiment [39,40,41,42].

Mitochondria play a crucial role in cellular energy metabolism. Most intracellular ATP is produced by mitochondrial respiration [22]. When mitochondrial ATP production slows down, so does respiration, which serves as fuel. Therefore, the rate of ATP-related respiration can be approximated by the rate of oxygen consumption [38]. 

In our study, the rate of oxygen consumption by platelet mitochondria in the control group was 18%, whereas it was 33% in the study group. This means that a process of hyperoxia took place: a pathological condition caused by an increased level of oxygen supply to the tissue. This is probably related to the specific type of lipid emulsion used, namely one rich in omega-3, -6, or -9 fatty acids. However, further studies are needed to confirm this hypothesis. This may induce lipid peroxidation processes in the cell and cause excessive oxidation of cellular components, including cellular DNA. 

The extent of genomic DNA damage varied considerably in our study. Damage levels of 3.5% and more were observed. This proves that mitochondrial DNA was significantly damaged by the supply of molecular oxygen as a result of inflammatory processes in the cell. The applied method showed that even the smallest changes in oxygen metabolism in mitochondria and the analyzed liver parameters were related. Typical symptoms of hyperoxia are headaches, excessive sleepiness, general weakness, tachycardia, tachypnea, shortness of breath, impaired consciousness, and visual disturbances.

Oxygen also affects the inflammatory response. Experimental models and studies in volunteers and patients show that superoxide (and hypoxia) can trigger proinflammatory and anti-inflammatory responses that have both protective and harmful effects [43,44].

In recent years, oxidative stress has been shown to play a key role in the development and maintenance of inflammation and contribute to the pathophysiology of many devastating human diseases [45]. OS refers to an imbalance between the production of ROS and the ability of endogenous antioxidant systems to clear ROS, with ROS overcoming antioxidant capacity [46,47]. The formation of ROS is not thought to be harmful under normal physiological conditions and is safely controlled by antioxidant mechanisms. ROS are important triggers and modulators of cell signaling and thus cell behavior. When this balance is slightly tilted in favor of ROS, continuous low-level oxidative damage occurs in the biological system [47]. The impairment of antioxidant defense mechanisms may lead to an increase in free radical-induced tissue damage. Oxidative stress plays a key role in the development of hypoxic diseases. Thus, excessive ROS are involved in irreversible damage to cell membranes, DNA, and other cellular structures through the oxidation of lipids, proteins, and nucleic acids [46,47].

Oxidative stress has been shown to have multidirectional effects in these conditions. Molecular oxygen is the major substrate of aerobic metabolism. Therefore, knowledge of cellular oxygenation is central to a detailed understanding of the cellular metabolic response to a particular treatment or manipulation. Studies conducted with the use of the Fpg protein show that oxidized compounds are novel substrates for this protein [39,40,41,42]. In particular, guanine damage plays a key role in the formation and repair of ethene and propane damage in the patient samples studied. 

We observed that the percentage of molecular oxygen depends on the type of lipid emulsion supplied. With the duration of parenteral nutrition, the percentage of genomic DNA damage tends to decrease after Fpg protein treatment and the percentage of molecular oxygen in cells increases by using the oxygen consumption assay method. However, it is unclear whether parenteral nutrition has a direct effect on genomic DNA damage and molecular oxygen levels in cells over the course of treatment.

This research is unique and innovative because it is the first time both methods have been used, namely Fpg protein digestion of genomic DNA from patients’ blood and the application of the method of molecular oxygen consumption in mitochondria, which are excellent markers of oxidative stress, helpful in determining the process of lipid peroxidation (fat oxidation) in which ethene and propane DNA derivatives are formed and whose end product is malondialdehyde or trans-4-hydroxy-2-nonenal. Fpg is a protein from the group of repair glycosylases. These enzymes are markers for altered oxidized bases in nucleic acids that are formed during oxidative stress in cells. This is particularly important for parenteral nutrition, where various types of lipid derivatives from the omega-3, -6, and -9 groups are used.

We believe that the obtained results can be of great importance for further pharmaceutical studies and their practical application. For example, TPN is a life-saving therapy for patients with intestinal failure. This manuscript may be helpful to determine the dependencies between the liver enzymes ALT and AST which often increase after long-term TPN in relation to the rate of oxygen consumption in platelet mitochondria in patients receiving long-term TPN with the degree of oxidative stress induced by lipid emulsions. This may help explain their role in cellular energy metabolism and the changes in the liver based on the percentage of damaged genomic DNA.

## 6. Conclusions

It is unclear whether parenteral nutrition has a direct effect on genomic DNA damage and molecular oxygen levels in cells over the course of treatment. The products of ROS-dependent lipid metabolism are also generally considered to promote inflammation and are observed at higher levels in disease. The complex interactions between lipids and the immune system may be amplified during the disease process and, together with oxidative stress, constitute an important factor in disease processes, but is not yet fully understood. The effects may depend on various factors, such as the patient’s health status, underlying disease, the duration and type of parenteral nutrition, and other medical interventions. It is important to monitor patients receiving parenteral nutrition closely for adverse effects and to manage any potential risks appropriately. It would also be important to consider other factors that may affect ALT/AST levels, such as age, sex, medical history, and medication use. This may facilitate the development of personalized and precise parenteral nutrition programs to reduce the development of inflammation and tailor interventions to the patient’s physiological state to reduce proinflammatory and prooxidant pathways.

## Figures and Tables

**Table 1:** Age and time of TPN for the whole group and by gender.

	Total		Female (*n* = 44)		Male (*n* = 42)		t_df=84_	*p*-Value *
	M	SD	M	SD	M	SD		
Age	56.67	17.42	57.48	15.30	55.83	19.56	0.435	0.664
TPN [years]	5.69	6.75	5.25	6.64	6.14	6.92	−0.611	0.543

M—mean, SD—standard deviation, df—degrees of freedom, * Student’s *t*-test.

**Table 2:** The volume of lipid emulsions and the number of grams of lipids in the TPN for the whole group and by gender.

	Total		Female (*n* = 44)		Male (*n* = 42)		t_df=84_	*p*-Value *
	M	SD	M	SD	M	SD		
Lipid emulsion/week (mL)	766.00	340.00	697.95	349.48	838.10	318.11	−1.942	0.056
Lipid/week (g)	151.00	61.92	136.86	57.56	166.79	63.28	−2.296	0.024

M—mean, SD—standard deviation, df—degrees of freedom, * Student’s *t*-test.

**Table 3:** AST, ALT, and CRP results for the whole group and by gender.

	Control Group		TPN Group				
	Mean	SD	Mean	SD	t *	df	*P*
AST [U/L]	24.24	15.87	48.44	34.78	5.423	170	0
ALT [U/L]	26.6	13.3	30.26	20.06	2.18	170	0.031
CRP [mg/L]	4.24	4.98	10.23	15.18	3.641	170	0

SD—standard deviation, df—degrees of freedom, * Student’s *t*-test.

## Data Availability

The raw data supporting the conclusions of this article will be made available by the authors without undue reservation.
